# Autotaxin and Breast Cancer: Towards Overcoming Treatment Barriers and Sequelae

**DOI:** 10.3390/cancers12020374

**Published:** 2020-02-06

**Authors:** Matthew G. K. Benesch, Xiaoyun Tang, David N. Brindley

**Affiliations:** 1 Discipline of Surgery, Faculty of Medicine, Memorial University of Newfoundland, St. John’s, NL AlB 3V6, Canada; 2 Cancer Research Institute of Northern Alberta, Department of Biochemistry, Faculty of Medicine and Dentistry, University of Alberta, Edmonton, AB T6G 2S2, Canada; xtang2@ualberta.ca

**Keywords:** lysophosphatidic acid, lipid phosphate phosphatases, GLPG1690, chemoresistance, radiotherapy, metastasis, tumor microenvironment

## Abstract

After a decade of intense preclinical investigations, the first in-class autotaxin inhibitor, GLPG1690, has entered Phase III clinical trials for idiopathic pulmonary fibrosis. In the intervening time, a deeper understanding of the role of the autotaxin–lysophosphatidate (LPA)–lipid phosphate phosphatase axis in breast cancer progression and treatment resistance has emerged. Concordantly, appreciation of the tumor microenvironment and chronic inflammation in cancer biology has matured. The role of LPA as a central mediator behind these concepts has been exemplified within the breast cancer field. In this review, we will summarize current challenges in breast cancer therapy and delineate how blocking LPA signaling could provide novel adjuvant therapeutic options for overcoming therapy resistance and adverse side effects, including radiation-induced fibrosis. The advent of autotaxin inhibitors in clinical practice could herald their applications as adjuvant therapies to improve the therapeutic indexes of existing treatments for breast and other cancers.

## 1. Introduction—History of Breast Cancer, Current Management, and Remaining Challenges

Breast cancer is believed to be the oldest documented cancer, described in an Egyptian scroll dating to 1700 BCE as a bulging mass for which no cure was possible [1]. Writings of Hippocrates and Galen describe crab-like lesions (*karkinoma*) of the breast with swollen blood vessels and a hardened, matted surface, from which we get the word, “carcinoma” [2]. These works also describe a “black bile” discharge from breasts, which we now recognize as signs of symptomatic breast cancer [3,4]. Virtually no progress in treatment was made until the first mastectomies were performed in the 1750s. Once the concepts of lymphatic spread and metastasis were understood, the radical mastectomies of the early 20th century gave way to lumpectomies and sentinel lymph node biopsies. The 20th century saw cancer treatment develop into a trimodal entity—surgery, the “cold knife”, the remove the cancer with a margin of healthy tissue; radiotherapy, “the hot knife”, to eradicate any remaining cells within the surgical field; and chemotherapy, to both eliminate any circulating cancer cells and prevent local reoccurrence.

Clinically, the treatment of breast cancer has made incredible progress, but challenges remain. Examining SEER (Surveillance, Epidemiology and End Results) data from the United States, among the largest cancer epidemiology repositories in the world, overall 5-year survival has risen from 74.6% to 92.4% from 1975 to 2015 [5]. However, the gains made in survival do not equally apply to all groups of breast cancer at disease presentation. Survival for regional breast cancer has increased from 52% to 85%, whereas survival from distant or metastatic disease has only marginally improved from 13% to about 20% in 30 years [5,6]. Because treatment of metastatic disease rests largely on chemotherapeutic interventions, discovering how to overcome either inherent or acquired chemoresistance mechanisms is necessary to both prolong remission and increase survival rates.

Chemotherapeutic interventions for breast cancer depend primarily on the estrogen and progesterone hormonal status of the tumors. Whether localized or disseminated, the treatment of choice is based on endocrine therapies, including selective estrogen receptor modulators (SERMs), aromatase inhibitors (AIs), and selective estrogen receptor down-regulators (SERDs) [7]. A third receptor, HER2/neu (human epidermal growth factor receptor 2), is overexpressed in up to 20% of breast cancers and it is targeted with receptor blockers including trastuzumab, pertuzumab, neratinib, and lapatinib [8]. Breast cancers that express none of these receptors are labeled triple negative, accounting for 10%–20% of all cases, and typically they are poorly differentiated with higher proliferation rates compared to hormone receptor-positive cancers. Their treatment depends on cytotoxic taxane-, anthracycline-, and platinum-based regimens that target cell-cycle progression. In approximately 20% of these patients, tumors will completely regress after such therapy (complete pathological response), but for those patients that do not achieve this level of response, the risk of reoccurrence and death from metastases are many fold more than for those with hormone-positive cancers [9,10]. Overall, 90% of breast cancer patients who develop metastatic disease become resistant to their chemotherapy regimens [11].

Despite the high heterogeneity and a plethora of molecular signatures, there are common mechanisms of chemotherapy resistance in triple-negative breast cancers, which have also become recognized in recent years to be associated with treatment failure against hormonal and HER2/neu targeted treatments [10,12]. By no means exhaustive, three of these mechanisms of interest in breast cancer research are ATP-binding cassette (ABC) transporters, cancer stem cells, and microRNAs. Briefly, ABC transporters, or multi-drug resistance transporters, use ATP to export a host of chemotherapeutic agents from cancer cells. Of the 49 known transporters in humans, three are most often implicated in cancer therapy resistance: multidrug resistance protein 1 (MDR1 or ABCB1), multidrug resistance-associated protein 1 (MRP1 or ABCC1), and breast cancer resistance protein (BCRP or ABCG2) [13].

Next, cancer stem cells are a pluripotent sub-population of cells that are intrinsically resistant to chemotherapy due to their quiescence and dormancy. While cancer cells within a tumor are killed by treatment, these highly plastic survivors spawn cells with genetic adaptations permitting resistance to subsequent rounds of treatment [14]. Normally comprising 1% of the total tumor cell population, their fraction increases up to 30% with tumor progression following treatment failure [15].

MicroRNAs are a family of small non-coding single-stranded regulatory RNAs that bind to the 3′-untranslated region of target messenger RNAs to act as post-transcription downregulators of translation [16]. Cancer cells manipulate the balance of microRNAs to favor cell growth and survival, proactively adapting their expression profile to the evolving state of cancer progression [17]. Over 900 microRNAs have been characterized, and their role within breast cancer and treatment failure is reviewed elsewhere [16,18].

Perhaps the most fundamental paradigm shift in cancer research over the last decade has been the appreciation of the role of the tumor microenvironment. In the mid-2000s an average of one hundred papers per year appeared in PubMed on this subject, whereas the number of publications in 2019 alone is over four thousand. The tumor microenvironment describes the paracrine nature of interactions among cancer cells and other cell types within tumor stroma and their signaling mediators that are subverted towards tumor propagation. An abetting microenvironment is among the most prominent of the emerging hallmarks of cancer [19,20,21]. The secretory milieu of inflammatory mediators by cancer associated fibroblasts, immune cells, endothelial cells, and mesenchymal stromal cells is subverted by the ever adaptive tumor to overcome impediments to its growth, spread, and survival against treatment [22]. As drivers of resistance, cancer stem cells are protected by the tumor microenvironment through adaptation to nutritional, metabolic, and oxygen deprivation stresses [15].

Breast cancer has become the prototypical model for the tumor microenvironment, in part because its interactions with tumor stroma and adjacent breast tissue are complex and our understanding of these events is at an elementary stage [23]. Deconvoluting these interactions and developing selective pharmacological approaches to break the communications between cancer cells and stroma could have as great an impact on cancer therapy in the next ten years as immunotherapy has had over the last decade. The benefits of pharmacological inhibition of signaling from the tumor microenvironment should have therapeutic applications for many cancer types.

In this review, we will provide an updated summary on the role of the autotaxin– lysophosphatidate–lipid phosphate phosphatase (ATX–LPA–LPP) axis in cancer progression and its treatment with focus on breast cancer. We will briefly summarize historical research milestones on this axis, recent insights into the tumor microenvironment, and pre-clinical and clinical developments related to disruption of pathologic ATX–LPA–LPP signaling.

## 2. Overview of the ATX–LPA–LPP Axis and Its Historical Context

**Figure 1:**
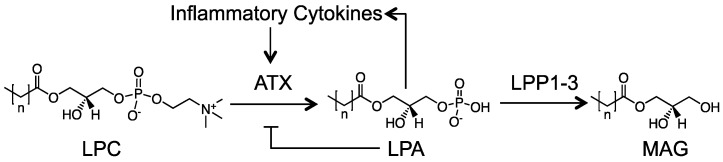
Overview of the lysophosphatidate (LPA) signaling axis. Extracellular LPA is produced from lysophosphatidylcholine (LPC) by the lysophospholipase D activity of autotaxin (ATX). LPA then signals through six known G-protein coupled LPA receptors to mediate its host of physiological and pathological effects. LPA is rapidly turned over by the ecto-activity of LPP1-3 into MAG (monoacylglycerol) and inorganic phosphate. In response to tissue damage, a feed-forward loop is established where inflammatory cytokines increase ATX production, overriding the natural feedback inhibition of LPA on ATX transcription.

Autotaxin (ATX) is a secreted lysophospholipase D that generates most of the extracellular lysophosphatidate (LPA) from hydrolysis of choline from lysophosphatidylcholine (LPC), which probably does not have direct signaling functions (Figure 1) [24]. This LPA then signals through at least six known LPA receptors (LPAR1-6) [25]. LPA is degraded by the ecto-activities of three lipid phosphate phosphatases (LPP1-3) [26]. This process is very rapid and the half-life of LPA in the circulation is about 1 min [27]. In addition to attenuating LPA signaling by degrading extracellular LPA, LPP1 attenuates signaling downstream of LPA and protease-activated receptors. This effect requires the catalytic activities of LPP1 [26,28]. Signaling by LPA facilitates wound healing by increasing the migration and division of cells needed for tissue repair and angiogenesis [29]. After injury, ATX secretion is stimulated following the release of inflammatory cytokines [29,30]. This process overcomes the normal feedback inhibition of LPA on the transcription of ATX. Increased synthesis of LPA then stimulates the synthesis of COX-2 and more inflammatory cytokines, which causes additional ATX secretion in a feed forward cycle (Figure 1) [30,31,32]. LPA increases innate immune responses [30] and promotes lymphocyte extravasation and conversion of monocytes to macrophages, which maintains immune homeostasis [33]. Inflammation resolves when the tissue is repaired and ATX secretion decreases accordingly [29]. Further details on the biology of this ATX–LPA–LPP axis are well reviewed elsewhere [24,26,30,34].

As in the case of most tumor biology, cancer initiation and progression involve subversion of physiological cellular processes. Often, our understanding of cellular biology begins through study of maladaptive cancer biology. The ATX–LPA–LPP axis fits into this framework. ATX was first identified as an “autocrine motility factor” found in cell culture medium of melanoma cells in 1992 [35]. Later independent work in 1995 showed that LPA among lysophospholipids could uniquely exert cell signaling events through unique receptors in breast cancer cells and thus could be a potential target for therapy [36]. LPA signaling was linked to breast cancer cell proliferation in 1999 [37]. In 2002, ATX was identified as the primary enzyme that produces extracellular LPA, and that the effects of ATX on cellular processes are mediated through LPA signaling [38,39]. Studies in the early 2000s demonstrated that global LPP expression is decreased in cancer cells, and that overexpression of LPP3 decreases the growth, survival, and tumorigenesis of ovarian cancer cells [40]. Overall, it was established primarily in vitro by the mid-2000s that increased LPA signaling through overall increased ATX activity and LPAR expression with concomitant decreases in catalytic LPP activity creates a pro-growth and pro-survival state for cancer cells. Finally, seminal work in 2009 demonstrated a causal role for ATX and LPARs in breast tumorigenesis using a mouse mammary tumor virus (MMTV)-driven breast cancer murine model. In this work, transgenic mice with wild-type LPARs 1–3 or ATX had higher incidence of tumor onset and metastases compared to controls [41]. In 2012 these findings were shown to be clinically relevant since increased expressions of ATX in the stroma and LPAR3 in epithelial cells are associated with aggressiveness of human breast cancer in women [42]. ATX concentrations correlate with invasiveness [24,43,44] and the ATX gene (*ENPP2*) is one of the 40–50 most up-regulated genes in metastatic tumors [45,46,47].

Over the past decade much investigation has led to significant insights into the mechanistic aspects of autocrine and paracrine signaling effects of LPA, and their context within the overall tumor microenvironment architecture. This work has occurred both in tandem and parallel with the evolution of pharmacological inhibition of the ATX–LPA–LPP axis. With such inhibitors now in clinical trials, medical use of these treatments will become a reality in the 2020s for cancers and other chronic inflammatory conditions.

## 3. Maladaptive Effects of Excessive ATX Secretion and LPA Signaling in Inflammation, Fibrosis, and the Tumor Microenvironment

As previously discussed, physiological upregulation of ATX occurs as part of acute inflammation in wound healing and then dissipates after tissue repair is completed. If inflammation is not resolved, chronic activation of ATX-LPA-inflammatory signaling and the wound healing response becomes maladaptive [30,32] in diseases such as pulmonary fibrosis, cirrhosis, rheumatoid arthritis, inflammatory bowel disease, and cancers [24,48]. Many inflammatory conditions are accompanied by fibrosis, a process driven through LPAR1 signaling and further mediated through inflammatory cytokines [49,50,51,52,53,54,55,56,57,58,59,60,61]. This is why an ATX inhibitor, GLPG1690 [62] and an LPAR1 antagonist (BMS986020) [63] attenuated idiopathic pulmonary fibrosis in Phase IIa clinical trials. GLPG1690 is a first in-class drug that has entered Phase III trials for idiopathic pulmonary fibrosis [64].

In some cancers, such as melanoma, glioblastoma, and thyroid, ATX is secreted directly by the cancer cells [65,66]. This increased ATX activity is hijacked in cancers (wounds that do not heal) to promote a chronic inflammatory state leading to the secretion of a milieu of pro-growth and survival inflammatory mediators [21,67,68]. Tumor-promoting inflammation and decreased acquired immune responses are “hallmarks” of cancer [21,24,69]. In addition, chronic LPA signaling enables cancer cells to evade the immune system [30,32,70]. Recent work shows that this involves increased signaling through LPAR5 on CD8+ T-cells and blocking of T-cell antigen receptor responses [71]. LPA also increases vascular endothelial growth factor (VEGF) production, which stimulates the angiogenesis needed for tumor growth [72]. This increased LPA signaling also upregulates expression of cancer stem-cell genes *OCT4*, *SOX2,* and *ALDH1,* thereby improving tumor survival after treatment via repopulation of cancer cells from differentiated cancer stem cells [73]. The ATX gene (*ENPP2*) is the second most upregulated gene in cancer stem cells derived from HMLER breast cancer cell cultures treated with paclitaxel, and the LPP2 gene (*PPAP2C/PLPP2*) is the most downregulated, favoring an LPA-enriched environment [74]. ATX upregulation also correlates to dysregulated microRNA regulation, favoring a pro-tumorigenic state [75,76]. A thorough review on the micoRNA regulation of the ATX-LPA signaling axis has recently been published [77].

**Figure 2:**
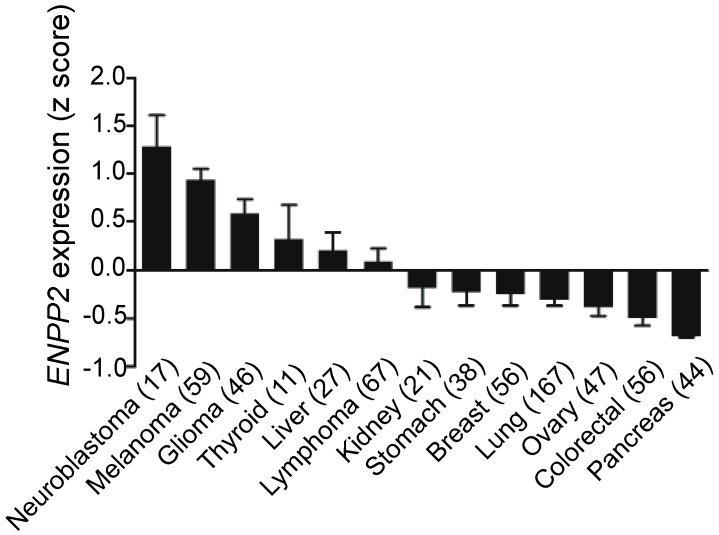
Overview of ATX mRNA expression across cell lines. Breast cancer cell lines express extremely low ATX mRNA relative to other cancer types. Numbers in parentheses indicate number of cell lines. Results from cBioPortal (www.cbioportal.org) [86,87].

**Figure 3:**
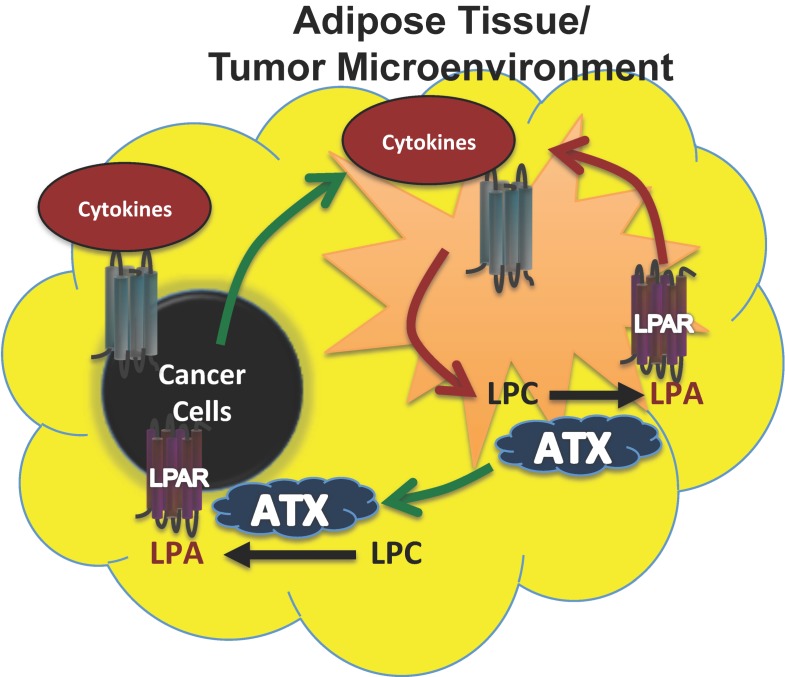
Model for ATX production in breast cancers where the cancer cells produce little ATX themselves. In breast cancers, a paracrine model of ATX secretion is possible because adipose tissue secretes high levels of ATX, whereas breast cancer cells normally produce negligible ATX. As the tumor grows, proinflammatory signals (cytokines) secreted by the tumor create an inflammatory environment within the surrounding adipose tissue and tumor microenvironment (green arrows). This signaling increases ATX secretion and LPA production, which in turn can establish an autocrine feed forward loop of increased cytokine signaling and ATX production (red arrows) within the adipose tissue and tumor microenvironment. Increased ATX and LPA production contribute to tumor progression (green arrows). Figure adapted and modified with permission from [31].

Breast cancer cells, however, produce little ATX [66,78,79,80] as do kidney, stomach, lung, ovarian, colorectal, and pancreatic cancer cells (Figure 2). In these cancers, the tumor microenvironment is instead the primary source of ATX. To support this, we showed that human breast tissue constitutively expresses about four time more ATX mRNA than breast tumors [31]. Furthermore, we also showed by immunohistochemistry that the tumor stroma expresses nearly three-fold more ATX protein than normal breast stroma in matched patient samples [31]. Combining these results with murine studies, we established a new model for understanding breast cancer in which inflammatory cytokines produced by breast tumors increase ATX secretion by breast adipocytes and tumor-associated fibroblasts (Figure 3) [29,31,80]. This amplifies the inflammatory cycle and promotes accumulation of inflammatory leukocytes in the inflamed adipose tissue [30,32]. Bi-directional signaling between breast tumors and surrounding adipose tissue through the ATX-LPA-inflammatory cycle (Figure 3) has been confirmed [81,82]. At the organism level, we showed this increased ATX activity is measurable in the plasma of mice with advanced breast cancer [29]. In human breast cancer, serum ATX has since been investigated a novel biomarker for nodal disease [83]. More recent studies demonstrate that ATX produced by platelets provides LPA for establishing a hospitable niche for metastatic cancer cell seeding, further enhanced by additional signaling mediated by ATX binding to cell surface integrins at the sites of metastases [84,85].

**Figure 4:**
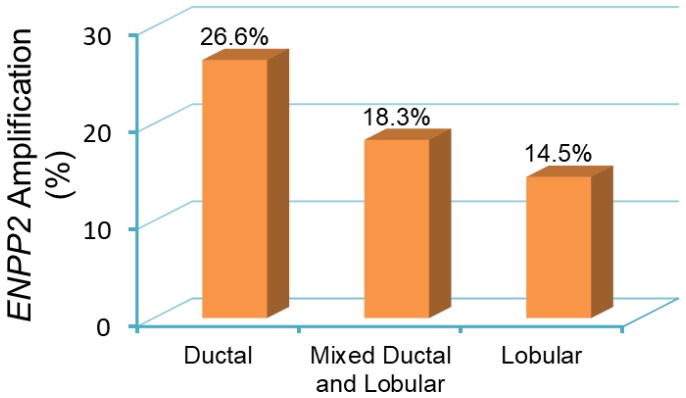
*ENPP2* amplification in primary breast tumor samples from patients [92]. Breast tumor samples are categorized as ductal, lobular, and mixed ductal and lobular carcinoma. The incidence of *ENPP2* amplification is 26.6% in ductal (441 out of 1660 cases), 18.3% in mixed ductal and lobular (40 out of 218 cases), and 14.5% in lobular (25 out of 172 cases) breast carcinomas. Data is obtained and analyzed from cBioPortal (www.cbioportal.org) [86,87].

**Figure 5:**
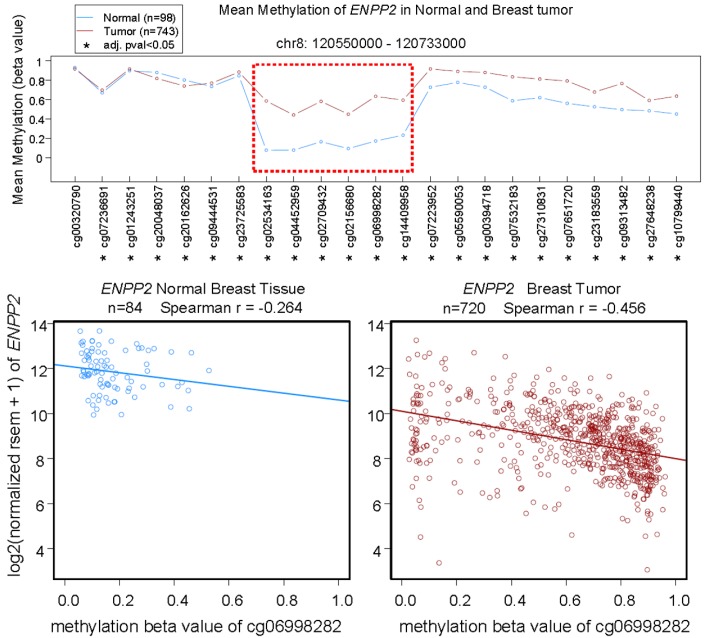
Top: Mean methylation (β values) of *ENPP2* in the displayed genomic region of normal breast tissue and breast tumors measured by Illumina HumanMethylation450 BeadChip. Horizontal axis lists the codes of HumanMethylation450 probes for each methylation site. Data are analyzed with Wilcoxon rank sum test, * adjusted *p* value < 0.05. The most different methylation level between normal and tumor tissue is highlighted by the red box. Bottom: Correlation of mean methylation of *ENPP2* at the site for probe cg06998282 and *ENPP2* expression in normal breast tissue and breast tumors. Gene expression is expressed in RSEM (RNA-Seq by Expectation-Maximization) format. Data from Wanderer (www.maplab.imppc.org/wanderer) [93].

With this complex interaction between cancer cells and the tumor stroma, it is now possible to understand the genomic alterations in ATX expression and post-transcriptional regulation. Federico and colleagues demonstrated that ATX copy number amplification is present in just under 20% of all patient breast tumor samples [88]. It is possible to demonstrate a similar trend by using results from a survey of over 2000 breast cancer samples, with amplification more likely to be found in ductal cancers (26.6%) compared to lobular cancers (14.5%) (Figure 4). Despite this, the ATX gene is overall more highly methylated in tumors compared to normal breast tissue (Figure 5), implicating that the functional pool of ATX in breast tumors depends on the induction of ATX expression in the tumor microenvironment (Figure 3). Post-transcriptionally, the RNA-binding protein ELVA-like protein 1 or human antigen R (HuR) enhances ATX mRNA stability in melanoma cells, and HuR protein expression increases in response to LPA [89]. High HuR levels are a prognostic factor for poor outcome in breast cancer [90]. Finally, breast cancer cells recruit ATX to their cell surface through β3 integrin binding to promote persistent directional cell migration [91]. Overall, the source of increased ATX in breast cancer is multifactorial, but it is likely to be heavily dependent on the ability of the cancer cell to manipulate the overall functional protein level stochastically and spatially.

The association between obesity, chronic inflammation, and cancer risk is no coincidence, and ATX/LPA signaling may be a central feature underpinning the relationship. About 40% of ATX in mice is produced by adipocytes, and this increases on feeding a high fat “human-type” diet [94,95]. In obese patients, increased ATX is associated with more visceral fat compared to non-obese patients [95]. ATX production increases in obesity, especially when adipose tissue is inflamed [24,30]. Inflamed adipose tissue increases the co-morbidities of insulin resistance, diabetes, dyslipidemia, hypertension, and atherosclerosis [96,97], and serum ATX levels positively correlates with insulin resistance in older obese adults [98]. These conditions are characterized by low plasma adiponectin concentrations [99] and LPA decreases adiponectin secretion [94]. ATX could contribute to the association of obesity with ~30% of breast cancers [100,101]. As an example, LPA signaling induces microvascular remodeling in chronic diet-induced obesity via CD36-mediated signaling, a cell surface glycoprotein associated with angiogenesis in ER-positive breast cancers [102]. Adipokine biology in breast cancer progression is an extensive area of investigation and is further reviewed elsewhere [103,104].

Overall, targeting the pro-inflammatory tumor microenvironment to combat failure of cancer treatment is a natural progression for ATX–LPA–LPP axis research. We have demonstrated that adipocyte-derived ATX is a key inflammatory regulator in breast cancer. By inhibiting ATX activity in a mouse model of breast cancer, plasma concentrations of TNFα and G-CSF by decreased ~10-fold [31]. The concentrations of >16 inflammatory cytokines/chemokines in the fat pad adjacent to the breast tumor were also decreased and this was accompanied by decreased infiltration of CD45+ leukocytes. We have also shown that inhibition of ATX activity [31,80] or increased LPA degradation by LPP1 [27,105,106] decreases breast tumor growth and metastases in mice.

## 4. Effects of LPP Expression in Cancers

The other side of the equation from the production of LPA by ATX is its rapid turnover by the LPPs, which also attenuate signaling downstream of LPA receptors and protease activated receptors [26]. The expressions of LPP1 and LPP3 (*PLPP1* and *PLPP3*, respectively) are decreased in lung, ovarian, and breast tumors [107,108,109]. This contributes to the increase in LPA concentrations in the tumors [110,111,112]. Low LPP1 mRNA expression (*PLPP1*) is one of 12 changes in mRNA that predicts poor survival in breast cancer patients [113]. We showed that increasing the low levels of LPP1 in breast cancer cells decreases cell division and blocks tumor growth and metastases in a mouse breast cancer model by ~80% [27]. Also, increasing LPP3 expression in ovarian cancer models blocks tumor growth [27,114].

Our recent work shows that the expression of LPP1 and LPP3 are decreased in all types of breast tumors compared to normal breast tissue [26,106]. Patients with the lowest levels of LPP1 expression had a poorer prognosis, but this effect was not significant for LPP3 [106]. To investigate the consequences of the effects of LPP1 in breast cancer, we increased its expression in MDA-MB-231 breast cancer cells, which decreased their ability to invade through Matrigel. This was accompanied by decreases in expression of MMP-1, -3, -7, -9, -10, -12, and -13, which are transcriptionally regulated by the AP-1 complex, a dimeric transcription factor consisting of cFOS, cJUN, Fos-Related Antigen 1 (FRA1), and other subunits [106]. Increasing LPP1 attenuated the induction of mRNA of MMP-1, -3, cFOS, and cJUN by epidermal growth factor (EGF) or TNFα, but increased FRA1. LPP1 expression also decreased the induction of protein levels for cFOS and cJUN in nuclei and cytoplasmic fractions by EGF and TNFα. Protein levels of cyclin D1 and D3 were also decreased by LPP1. Although FRA1 in total cell lysates or cytoplasm was increased by LPP1, nuclear FRA1 was not affected. The decreases in MMPs in xenograft MDA-MB-231/LPP1-overexpressing tumors was accompanied by increased collagen in the tumors and fewer lung metastases [106]. Conversely, knockdown of LPP1 in MDA-MB-231 cells increased the protein levels of MMP-1 and -3. Human breast tumors also have lower levels of LPP1 and higher levels of cJUN, cFOS, MMP-1, -7, -8, -9, -12, -13, cyclin D1, and cyclin D3 relative to normal breast tissue [106]. We concluded from this study that the low LPP1 expression in breast cancer cells is associated with high levels of cyclin D1/D3 and MMPs as a result of increased transcription by cFOS and cJUN [106]. Increasing LPP1 expression could provide a novel approach for decreasing transcription through AP-1, which could provide a strategy for decreasing tumor growth and metastases.

In contrast to the decreased expressions of LPP1 and LPP3 in breast, lung and ovarian tumors, mRNA concentrations for LPP2 (*PLPP2*) are increased [26]. A genomic screen between normal and transformed mesenchymal stem cells and cancer cells showed that LPP2 is elevated in several cancer cell lines including MCF7, SK-LMS1, MG63, and U2OS [115]. We showed that increasing LPP2 expression in fibroblasts stimulates cell division [116]. The increased LPP2 expression in cancer cells is part of the transformed phenotype and it facilitates anchorage-dependent cell growth [115]. Our unpublished work shows that knockout of LPP2 in MDA-MB-231 breast cancer cells decreases tumor growth by ~70% in an orthotopic mouse breast model.

## 5. Effects of LPA on the Efficacy of Chemotherapy

**Figure 6:**
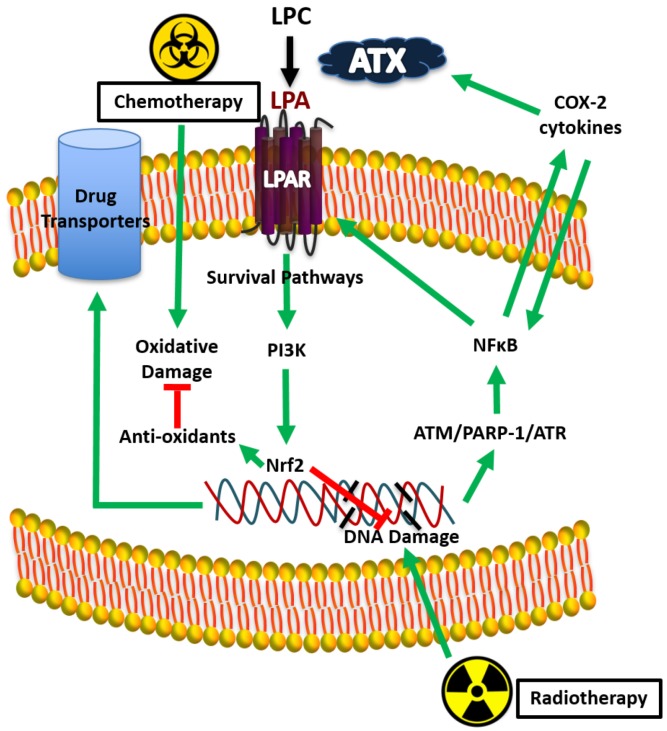
Proposed mechanism for lysophosphatidate (LPA) signaling and therapy resistance by Nrf2 activation.

LPA decreases the killing of breast cancer cells by paclitaxel [78], tamoxifen [117], and doxorubicin [118], which are major therapeutics for different types of breast cancer. Axiomatically, ATX inhibition increased the efficacy of doxorubicin in decreasing breast tumor growth and metastases in mice [119,120]. This LPA effect depends on the activation of LPAR1, which stabilizes the transcription factor, Nrf2 (Figure 6). This leads to activation of the anti-oxidant response element, which stimulates the synthesis of anti-oxidant proteins and multi-drug resistance transporters [118]. These changes protect cancer cells by decreasing oxidative damage and by exporting chemotherapeutic drugs and toxic oxidation products from cancer cells.

## 6. Effects of LPA on the Therapeutic Outcomes from Radiotherapy (RT)

About 60% of breast cancer patients receive breast-conserving surgery (lumpectomy) followed by RT involving ~16 daily fractions of 2 to 2.65 Gy to the post-operated breast [121]. The RT-induced cytokine surge [122] produces fatigue in patients [123]. We determined the consequences of irradiating human breast adipose tissue. A single dose of 0.25 to 5 Gy activated the ATX-LPA-inflammatory cycle by increasing the production of ATX, LPAR1/2, COX-2 and multiple inflammatory cytokines [124]. These events resulted from RT-induced DNA damage, which activate ATM, ATR, PARP-1, and NF B (Figure 6) [124]. Inhibiting ATR, PARP-1, and NF B decreases the RT-induced activation of the ATX-LPA-cycle. Higher radiation doses to intestinal cells produced a similar signaling cascade [119].

We extended our work by using precision RT on a mammary fat pad in mice using a small-animal “image-guided” RT platform (SARRP) with integrated CT-imaging. This allows treatment-planned RT to the tumor and fat pad while minimizing peripheral tissue damage. A single dose of RT increased plasma ATX concentrations. This result is remarkable as RT was focused only on one of the mammary fad pads [125]. There was no significant effect of one dose of RT on plasma concentrations of IL-6 and TNFα, but three fractions of RT substantially increased these inflammatory cytokines [125]. A similar increase was observed after three fractions of RT for VEGF, G-CSF, CCL11, and CXCL10 in the irradiated adipose tissue [125]. We also showed that one and three fractions of RT increase Nrf2 expression [125], which increases the synthesis of numerous proteins that attenuate oxidative damage and promote DNA repair, including the glutamate–cysteine ligase catalytic subunit, which helps to protect cell from radiation-induced damage [126,127]. In fact, Nrf2 blockade was proposed as a target for increasing the efficacy of RT [128]. These effects of multiple fractions of RT probably depend on the cumulative DNA and tissue damage. A similar augmentation of inflammation is expected in breast cancer patients when their breasts are treated with ~16 fractions of RT. Increased ATX production is an early event in response to RT and consequent LPA signaling is probably responsible for the subsequent increase in cytokine production.

Repeated activation of the ATX-LPA-inflammatory cycle should decrease the efficacy of RT by stimulating a wound healing response [129,130,131]. First, RT-induced increases in expressions of ATX and activation of LPAR2 decrease cancer cell death by depletion of the pro-apoptotic protein, Siva-1 [129]. However, apoptosis is not a major primary consequence of RT in breast cancer. Instead, human solid tumor-derived cell lines, including breast cancer cells, typically undergo some form of cytostasis (senescence or polyploid giant-cell formation) after RT [89]. A second consequence of RT is the stimulated expression of ATX and LPAR1, which increase Nrf2 expression resulting in protection from RT-induced damage (Figure 6) [118,125]. Thus, ATX inhibition with BrP-LPA or PF-8380 increased the sensitivity of heterotopic glioblastomas to RT in mice [132,133]. These compounds will not reach the clinic, but GLPG1690 is in Phase III trials. We showed that GLPG1690 decreases the proliferation of breast cancer cells in irradiated tumors [120], which supports the case for blocking LPA signaling to improve the efficacy of RT. In addition, blocking LPA signaling should decrease the co-morbidity of fibrosis.

## 7. LPA Signaling and Radiation Fibrosis Syndrome

Fibrosis is a common morbidity associated with RT such that RT for head and neck, thoracic, and pelvic cancers is restricted because of this [134]. Collectively, the constellation of symptoms from RT fibrosis is termed Radiation Fibrosis Syndrome [135]. RT to the whole breast after lumpectomy results in Grade 1 fibrosis in ~27% of patients at 3 years and Grade 2 fibrosis in ~1% [136]. Fibrosis increases over decades [137,138] and it requires both cosmetic correction in severe cases and psychological care [139]. RT-fibrosis also contributes to lymphedema, which results in impaired function and decreased quality of life [140]. There is inevitably some exposure of the lungs to irradiation during RT for breast cancer treatment, and lung fibrosis proceeds for up to 10 years [137,138]. Although the resulting loss of ~10% of lung function may not be clinically significant, it could be avoided. LPAR1 activation drives fibrosis in liver and lungs [49,50,51,52,53,54,55,56,57,58,59,60,141] and it is likely to drive RT-induced fibrosis [30,124,142].

We studied if dexamethasone (DEX), which is commonly used in cancer patients to decrease sequelae of inflammation, could decrease RT-induced fibrosis. We discovered that DEX attenuates LPA signaling by decreasing the expression of ATX and LPAR1 and by increasing LPP1 expression in human adipose tissue and in irradiated fat pads in mice [142]. These actions were accompanied by decreased production of multiple inflammatory cytokines. Likewise, DEX in our unpublished studies decreased the expression of LPAR1, IL-6, and TNFα in addition to increasing LPP1 expression in irradiated mouse breast tumors. This coordinated decrease in LPA signaling explained why treating mice with DEX during the 5 fractions of 7.5 Gy to a mammary fat pad in these studies decreased RT-induced fibrosis by ~70% after 7 weeks in the irradiated fat pad. DEX also attenuated fibrosis by ~70% in the underlying lungs that were exposed to radiation during administration of RT to the breast. This result is compatible with other studies where DEX attenuates RT-induced inflammation and lung fibrosis [143], but the present work is the first to link this to the actions of ATX and LPA signaling on breast fibrosis.

## 8. Conclusions

The present review summarizes current knowledge as to how activation of the ATX-LPA-inflammatory cycle promotes not only tumor growth, but also decreases the efficacies of various chemotherapies used to treat breast cancer. The other mainstay of breast cancer treatment is RT, but it sometimes fails to eliminate residual cancer cells and it can produce adverse side effects, such as fibrosis. RT “kills” cancer cells in part by damaging their DNA. This, together with cell debris and released proteins, causes inflammation, which in turn can enhance the immunologic elimination of cancer cells [144,145]. Although inflammation can be a key component of RT, we propose that persistent activation of the ATX-LPA-inflammatory cycle becomes maladaptive. This is because persistent LPA signaling and inflammation can increase the immune evasion of cancer cells. In addition, LPA protects cancer cells from RT-induced damage and this involves increased transcription through Nrf-2. Furthermore, LPA signaling through LPAR1 promotes fibrosis which is a long-term adverse side effect of RT.

Breast cancer has emerged as the prototypical cancer whereby ATX from the surrounding cells in the tumor microenvironment produce the bulk of LPA that drives cancer cell therapy resistance and spread. Most current chemotherapeutic regimens target the cancer cell directly but acquired treatment resistance remains a major clinical barrier. This failure is largely driven by the healing inflammatory milieu provided by the microenvironment that is not blocked or tampered down during treatment. As an adjuvant treatment, inhibitors of the ATX–LPA–LPP axis could act as a novel defense against acquired treatment resistance. With breakthroughs in understanding of the biology of the microenvironment, a new age of chemotherapy sensitizers that disrupt cancer cell communication with its host tissues, and in turn expose cancer cells to the immune system, should become standard of care. Such advancements would lead to chronic management, and even cure, for new populations of cancer patients.

At present, there is no clinical cancer therapy regimen that involves blocking ATX or LPA signaling. With the advent of ATX inhibitors or LPA receptor antagonists into the clinic to treat idiopathic pulmonary fibrosis, it is now feasible to test whether these approaches could improve the efficacies of chemotherapy and RT, while also alleviating the side effects of RT-induced fibrosis.
